# Erythropoietin Enhances Post-ischemic Migration and Phagocytosis and Alleviates the Activation of Inflammasomes in Human Microglial Cells

**DOI:** 10.3389/fncel.2022.915348

**Published:** 2022-06-24

**Authors:** Eren Arik, Ole Heinisch, Michaela Bienert, Lara Gubeljak, Alexander Slowik, Arno Reich, Jörg B. Schulz, Thomas Wilhelm, Michael Huber, Pardes Habib

**Affiliations:** ^1^Department of Neurology, Medical Faculty, RWTH Aachen University, Aachen, Germany; ^2^Institute of Molecular and Cellular Anatomy, Medical Faculty, RWTH Aachen University, Aachen, Germany; ^3^Department of Anatomy and Cell Biology, Medical Faculty, RWTH Aachen University, Aachen, Germany; ^4^JARA-BRAIN Institute of Molecular Neuroscience and Neuroimaging, Forschungszentrum Jülich GmbH and RWTH Aachen University, Aachen, Germany; ^5^Institute of Biochemistry and Molecular Immunology, Medical Faculty, RWTH Aachen University, Aachen, Germany

**Keywords:** ischemia-reperfusion, inflammation, EPO, ROS, NLRP1, NLRP3, NLRC4, AIM2

## Abstract

Recombinant human erythropoietin (rhEPO) has been shown to exert anti-apoptotic and anti-inflammatory effects after cerebral ischemia. Inflammatory cytokines interleukin-1β and -18 (IL-1β and IL-18) are crucial mediators of apoptosis and are maturated by multiprotein complexes termed inflammasomes. Microglia are the first responders to post-ischemic brain damage and are a main source of inflammasomes. However, the impact of rhEPO on microglial activation and the subsequent induction of inflammasomes after ischemia remains elusive. To address this, we subjected human microglial clone 3 (HMC-3) cells to various durations of oxygen-glucose-deprivation/reperfusion (OGD/R) to assess the impact of rhEPO on cell viability, metabolic activity, oxidative stress, phagocytosis, migration, as well as on the regulation and activation of the NLRP1, NLRP3, NLRC4, and AIM2 inflammasomes. Administration of rhEPO mitigated OGD/R-induced oxidative stress and cell death. Additionally, it enhanced metabolic activity, migration and phagocytosis of HMC-3. Moreover, rhEPO attenuated post-ischemic activation and regulation of the NLRP1, NLRP3, NLRC4, and AIM2 inflammasomes as well as their downstream effectors CASPASE1 and IL-1β. Pharmacological inhibition of NLRP3 via MCC950 had no effect on the activation of CASPASE1 and maturation of IL-1β after OGD/R, but increased protein levels of NLRP1, NLRC4, and AIM2, suggesting compensatory activities among inflammasomes. We provide evidence that EPO-conveyed anti-inflammatory actions might be mediated via the regulation of the inflammasomes.

## Introduction

Neuroinflammation is initiated in response to multiple endogenous and exogenous stimuli and has been implicated to affect the severity and progression of various neurodegenerative diseases and brain injuries including Alzheimer’s disease, Parkinson’s disease, multiple sclerosis as well as stroke (Iadecola and Anrather, 2011; Heneka et al., 2014; Anrather and Iadecola, 2016; Jayaraj et al., 2019). The latter is one of the leading causes of mortality and long-term disability worldwide and is characterized by a sudden decrease in blood flow to the brain resulting in neurological impairments (Hackett et al., 2000; Clarke et al., 2002). The vast majority of strokes are ischemic in nature and occur due to an occlusion of a cerebral artery leading to a rapid deprivation of both oxygen and nutrients in the affected brain region (O’Donnell et al., 2010). Following acute ischemic stroke (AIS), a plethora of complex interlinked molecular and cellular mechanisms to resolve cell stress and maintain tissue homeostasis are activated and may result in necrotic or apoptotic cell death (Doyle et al., 2008; Khoshnam et al., 2017; Tuo et al., 2022). Besides bioenergetic failure, excitotoxicity, cytotoxicity and oxidative stress, neuroinflammation in particular plays a crucial role in the pathophysiology of AIS and thus represents a potential target for neuroprotective and neurorestorative therapeutic approaches.

A promising agent to confer cytoprotection after AIS is the hematopoietic growth factor erythropoietin (EPO), one of the most successful and best-researched biopharmaceuticals in history (Simon et al., 2019). Since its approval by the American Food and Drug Administration (FDA) in 1989 for patients suffering from chronic renal insufficiency, three decades of research demonstrated that the pleiotropic EPO also exerts anti-apoptotic and anti-inflammatory effects in different neurological diseases including stroke (Grasso et al., 2002; Fisher, 2003; Kumral et al., 2004; Sättler et al., 2004; Lu et al., 2005; Kanaan et al., 2006; Habib et al., 2019a). A systematic review and meta-analysis of EPO in experimental stroke highlighted a reduction of infarct volumes by 30% and an improvement of neurobehavioral outcomes by 40% in rodents after EPO administration following AIS (Jerndal et al., 2010). Although the suppressive action of EPO on the activation of the transcription factor NF-κB and the subsequent production of the pro-inflammatory cytokines TNF-α, IL-6, and IL-1β is well established, the precise mechanisms of EPO-mediated anti-inflammatory effects and its impact on immune cells in the brain remain to be clarified (Villa et al., 2003).

The post-ischemic inflammation of nervous tissue is orchestrated by both infiltrating and brain-resident immunocompetent cells such as microglia. These macrophage-like cells are prime responders to pathological conditions in the brain and are well recognized to exert a key role in the initiation and maintenance of neuroinflammation (Hanisch and Kettenmann, 2007). Upon stress/inflammatory conditions, microglial cells may adopt a reactive state, migrate toward and phagocytose dead cells/debris, and initiate a range of host defense mechanisms, including upregulation of a variety of pro- and anti-inflammatory cytokines/chemokines and can thus be either protective or harmful to their surrounding neuronal tissue (Hanisch and Kettenmann, 2007). Anti-inflammatory and neuroprotective effects of microglia include the promotion of glial scar formation, promotion of neuronal plasticity and the release of anti-inflammatory cytokines such as TGF-β and IL-10 (Gaire, 2021). However, especially IL-1β and IL-18 have been attributed a decisive role in post-ischemic apoptosis and pyroptosis and seem to be mainly secreted by microglia (Liu and Quan, 2018). The maturation of IL-1β and IL-18 and that of gasdermin D (GSDMD) is executed by intracellular multiprotein complexes termed as inflammasomes (Liu et al., 2016; de Vasconcelos and Lamkanfi, 2020). These multimeric molecules recognize either pathogen-associated molecular patterns (PAMPs) or danger-associated molecular patterns (DAMPs), leading to their assembly and activation (Fann et al., 2013a). They are composed of three main components: cytosolic pattern recognition receptors (PRR), an adaptor protein termed as apoptosis-associated speck-like protein containing a CARD (caspase recruitment domain) (ASC) and pro-CASPASE1 (Schroder and Tschopp, 2010; de Rivero Vaccari et al., 2016). Activation of the inflammasomes in turn promotes the activation of CASPASE1, which eventually generates the active form of IL-1β, pro-IL-18 and GSDMD and thereby induces and promotes a pro-inflammatory type of cell death called pyroptosis by incorporation of the N-terminal domain of GSDMD (GSDMD-N) into the cell membrane and thus forming membrane pores and triggering subsequent membrane rupture leading to the release of pro-inflammatory cytokines, such as IL-1β and IL-18 (Duewell et al., 2010; Fann et al., 2013a; Shi et al., 2015; Liu et al., 2016; Zhang et al., 2019). The NOD, LRR, and pyrin domain containing 3 (NLRP3) inflammasome is the most prominent and best characterized member of the inflammasome family and has been implicated as the main agent involved in neuroinflammation (Luo et al., 2019). However, accumulating data suggest that other inflammasomes such as the NACHT, LRR, and PYD domains-containing protein 1 (NLRP1), the absent in melanoma 2 (AIM2), and the NLR family CARD-containing domain 4 (NLRC4) inflammasome substantially contribute to both neuroinflammation and post ischemic brain damage independently of NLRP3 (Fann et al., 2013b; Denes et al., 2015; Barrington et al., 2017; Poh et al., 2019; Habib et al., 2020; Kim et al., 2020). Inhibition of inflammasomes has been reported to dampen neuroinflammation not only in neurons, astrocytes and vascular endothelial cells (Abulafia et al., 2009; Deroide et al., 2013; Fann et al., 2013b,^2014^; Denes et al., 2015) but also in microglia as well (Poh et al., 2019; Liu et al., 2021). Microglia express the EPO-receptor, however, the impact of recombinant human EPO (rhEPO) on microglial activation and the subsequent induction of inflammasomes after ischemia remains elusive (Shang et al., 2011, 2012).

Since the majority of research in microglia is performed with cell lines derived from rodents, we have analyzed the rarely characterized human microglial clone 3 (HMC-3) cells regarding their tolerance to ischemia and subsequently investigated the impact of rhEPO on cell viability, metabolic activity, oxidative stress, phagocytosis, migration, proliferation, as well as on the regulation of the NLRP1, NLRP3, NLRC4, and AIM2 inflammasome sensor proteins and the inflammasome downstreams.

We provide evidence that rhEPO not only mitigated ischemia-induced oxidative stress and pyroptosis and overall cell death but also enhanced metabolic activity, migration and phagocytosis of microglial cells. In addition, rhEPO attenuated post-ischemic the regulation of the NLRP1, NLRP3, NLRC4, and AIM2 inflammasome sensors as well as the activation of their downstream effectors CASPASE1 and IL-1β. Pharmacological inhibition of NLRP3 by MCC950 did not affect the activation of CASPASE1 and the maturation of IL-1β after oxygen-glucose-deprivation-reperfusion (OGD/R), but it increased protein levels of NLRP1, NLRC4, and AIM2, suggesting compensatory activities among the four canonical inflammasomes. Our data suggest that, among other mechanisms, EPO mediates its cytoprotective effect via the regulation of inflammasomes.

## Materials and Methods

### Human HMC-3 Microglial Cell Line

HMC-3 cells (ATCC: CRL-3304), originally generated by Prof. Tardieu in 1995 via SV-40-dependent immortalization (Dello Russo et al., 2018), were kindly provided by Prof. Cordian Beyer, (Institute of Neuroanatomy RWTH Aachen University) and utilized in our previous study (Voelz et al., 2021). If not stated otherwise the HMC-3 cells were cultured in Dulbecco’s modified Eagle’s medium (DMEM, Pan Biotech, Aidenbach, Germany) supplemented with 10% fetal bovine serum (FBS, Pan Biotech, Aidenbach, Germany) and 0.5% penicillin/streptomycin [P06-07100 (P/S, Pan Biotech, Aidenbach, Germany)] for a final concentration of 50 U/ml penicillin and 50 μg/ml streptomycin. Cells were maintained in a humidified incubator at 37°C and 5% CO_2_. HMC-3 cells were sub-cultured every 2 days at approximately 80% confluency by trypsinization (Trypsin/EDTA, Pan Biotech, Aidenbach, Germany). Regular PCR controls against gene markers of cell types used in our laboratory ensured the purity of our HMC-3 cells. One day prior to experiments the cells were detached by trypsinization with subsequent centrifugation [400 g, 5 min, room temperature (RT)]. The cell pellet was resuspended in DMEM, 5% FBS, 0.5% P/S (all Pan Biotech, Aidenbach, Germany) and seeded in well dishes for experiments and on coverslips for ICC.

### Oxygen-Glucose-Deprivation

To mimic ischemic stroke *in vitro*, we performed OGD as previously described (Habib et al., 2019a; Zeyen et al., 2020; Heinisch et al., 2021). One hour prior to the experiment recombinant human erythropoietin (rhEPO) [1 IU/ml] (Epoetin alfa, Hexal, Holzkirchen, Germany) or MCC950 [1 μM] (AdipoGen life sciences, Liestal, Switzerland) was added to the cells. Oxygen depletion was induced in our customized hypoxia chamber (Part#: C174, C21, BioSpherix, Parish, NY, United States) by flooding the chamber with 100% inert nitrogen gas until the oxygen concentration reached <1%. To ensure stable OGD-conditions we monitored oxygen levels, temperature and pressure within the chamber. An additional oxygen sensor was used to monitor the oxygen level in the media (#200001735, PreSens, Regensburg, Germany). An orbital shaker was placed beneath the hypoxia chamber at 30 rpm ensuring better O_2_ depletion in the media. The chamber itself was placed in a humidified incubator set to 37°C. Normoxia controls were kept at 37°C, 5% CO_2_ and atmospheric pressure.

### Cell Viability

Cell viability was measured using a lactate-dehydrogenase (LDH) assay according to the manufacturer’s protocol (LDH, CytoTox 96^®^ Non-Radioactive Cytotoxicity Assay, Promega, Walldorf, Germany). The release of stable cytosolic lactate dehydrogenase (LDH) indicates late apoptosis or necrosis of cells. The amount of released LDH was measured in the supernatant at 490–520 nm using a microplate reader (Tecan GmbH, Männedorf, Switzerland). Cells treated with lysis solution served as internal positive control, as previously shown (Habib et al., 2014). Cell viability findings were validated by staining HMC-3 cells 1:2 with Trypan Blue staining (#T8154, Sigma-Aldrich, Taufkirchen, Germany). Viable cells with an intact cell membrane show no uptake of trypan blue whereas damaged cells are stained blue. Cells were counted by an automated cell counter (Roche Innovatis Cedex XS, Basel, Switzerland).

### Metabolism

Cell metabolism was measured by CellTiter-Blue^®^ assay according to the manufacturer’s protocol (CellTiter-Blue^®^ assay, Promega, Walldorf, Germany). The reduction of blue resazurin to pink fluorescent resorufin requires an intact mitochondrial respiratory chain and can be observed only in metabolically active cells. The reduction of resazurin was observed in viable cells at 590 nm by a microplate reader (Tecan GmbH, Männedorf, Switzerland) as previously described (Habib et al., 2013).

### Western Blotting

Western Blot (WB) analysis was performed as previously described (Zeyen et al., 2020; Heinisch et al., 2021). In brief, cells were lysed in ice-cold radioimmunoprecipitation assay (RIPA) buffer with added proteinase and phosphatase inhibitors (complete, #1187358001, Roche, Basel, Switzerland). 30 μg of protein per lane was separated by SDS-PAGE followed by transfer onto a PVDF membrane (Roche, Basel, Switzerland) and blocking with 5% skim milk (#T145.3, Roth, Karlsruhe, Germany) in tris-buffered saline containing 0.05% Tween20 (#8076.4, Roth, Karlsruhe, Germany). After incubation with a primary antibody the membrane was incubated with the corresponding horseradish-conjugated secondary antibody, followed by visualization using ECL™ Plus Kit (Thermo Fisher Scientific, Waltham, MA, United States). β-Actin served as a loading control. Densitometric analysis was performed using ImageJ (NIH, Bethesda, MD, United States). A list of antibodies and their dilution is given in Table 1.

**TABLE 1:** List of antibodies used for Western blotting.

Antibody against	Company	Order-No.	Host	Dilution
β-Actin	Abcam, Cambridge, United Kingdom	Ab3280	Mouse	1:1000
HIF-1α	Novusbio, Centennial, CO, United States	NB100-479	Rabbit	1:1000
CAIX	Novusbio, Centennial, CO, United States	NB100-417	Rabbit	1:1000
NLRP1	Cell Signaling, Danvers, MA, United States	4990	Rabbit	1:1000
NLRP3	Thermo Fisher Scientific, Waltham, MA, United States	PA5-79740	Rabbit	1:1000
NLRC4	Millipore, Burlington, MA, United States	06-1125	Rabbit	1:1000
AIM2	Bioss, Woburn, MA, United States	Bs-5986R	Rabbit	1:1000
GSDMD	Cell Signaling, Danvers, MA, United States	39754	Rabbit	1:1000
Mouse IgG	GE Healthcare, Chicago, IL, United States	NXA931	Goat	1:4000
Rabbit IgG	GE Healthcare, Chicago, IL, United States	NA934	Goat	1:5000

### CASPASE1 Glo Assay

CASPASE1 activity was measured by providing a luminogenic CASPASE1 substrate called Z-WEHD-aminoluciferin (Caspase-Glo^®^ 1 Inflammasome Assay, Promega, Walldorf, Germany). Cleavage by active CASPASE1 results in generation of proprietary, thermostable, recombinant luciferase (Ultra-Glo™ Recombinant Luciferase) and subsequent generation of light, proportional to CASPASE1 activity. Proteasomal degradation was inhibited by MG-132. The luminosity was measured in a 96-well plate according to the manufacturer’s protocol in a microplate reader (Tecan GmbH, Männedorf, Switzerland).

### Enzyme-Linked Immunosorbent Assay

Enzyme-linked immunosorbent assay for detection of total IL-1β was performed according to the manufacturers’ protocol (ab46052 Human IL-1 beta ELISA Kit, Abcam, Cambridge, United Kingdom). Pre-coated 96-well strips supplied by the kit were loaded with 100 μl of cell supernatant, standard, positive and blank controls and incubated with an additional 50 μl of the detection antibody (Biotinylated anti-IL-1 beta, Human IL-1 beta ELISA Kit, Abcam, Cambridge, United Kingdom) for 3 h at RT. Next 100 μL enzyme-conjugated detection reagent (Streptavidin-HRP, Human IL-1 beta ELISA Kit, Abcam, Cambridge, United Kingdom) was added for 0.5 h at RT. Lastly, 100 μL substrate was added (Chromogen TMB Substrate Solution, Human IL-1β ELISA Kit, Abcam, Cambridge, United Kingdom) and the signal was measured after 20 min at 450 nm in a microplate reader (Tecan GmbH, Männedorf, Switzerland). ELISA for detection of pro-IL-1β was performed according to the manufacturer’s protocol (DLBP00, Human Pro-IL-1 beta/IL-1F2 Quantikine Elisa Kit, R&D Systems, Minneapolis, MN, United States). Pre-coated 96-well strips supplied by the kit were loaded with 200 μl of cell supernatant, standard, positive and blank controls and incubated with an additional 50 μl of assay diluent for 1.5 h at RT, followed by incubation with 100 μL of Human-Pro-IL-1β Antiserum (Human Pro-IL-1β Antiserum, Human Pro-IL-1 beta/IL-1F2 Quantikine Elisa Kit, R&D Systems, Minneapolis, MN, United States) for 0.5 h at RT. Next, 100 μL of Conjugate (Human Pro-IL-1β Conjugate, Human Pro-IL-1 beta/IL-1F2 Quantikine Elisa Kit, R&D Systems, Minneapolis, MN, United States) was added for 0.5 h at RT. Lastly, after washing once again 200 μL substrate was added and the signal was measured after 20 min at 450 nm in a microplate reader (Tecan GmbH, Männedorf, Switzerland). ELISA for detection of GSDMD was performed according to the manufacturers’ protocol (ab272463, Human GSDMD ELISA Kit, Abcam, Cambridge, United Kingdom). Pre-coated 96-well strips supplied by the kit were loaded with 50 μl of cell lysate, standard, positive and blank controls and incubated with an additional 50 μl of the antibody cocktail (Human GSDMD Capture Antibody and Human GSDMD Detector Antibody, Human GSDMD ELISA Kit, Abcam, Cambridge, United Kingdom) for 1 h at RT.

Next after washing 100 μL of TMB Development solution supplied with the kit was added for 10 min at RT. Lastly, 100 μL stop solution was added and the signal was measured at 450 nm in a microplate reader (Tecan GmbH, Männedorf, Switzerland).

### Phagocytosis

Phagocytic activity of HMC-3 microglial cells was evaluated by measuring the uptake of pHrodo™ green conjugated bioparticles of *Escherichia coli* (Thermo Fisher, Waltham, MA, United States) after OGD as previously described (Habib et al., 2014; Schölwer et al., 2020) with minor modifications. Medium used in the assessment of phagocytosis was changed to Live Cell Imaging Solution (A14291DJ, Life Technologies, CA, United States). 5 μM Cytochalasin D (#C2618, Sigma-Aldrich, Taufkirchen, Germany) served as a negative control whereas 1 μg/ml LPS [from Salmonella Minnesota, R596(Re) (TLRgrade™), ALX-581-008-L002, Enzo, Farmingdale, NY, United States) served as a positive control. For quantification the number of pHrodo™ green positive cells was counted as previously described (Schölwer et al., 2020).

### Immunocytochemistry

Immunocytochemistry (ICC) was performed as previously described [41]. After stimulation cells cultivated on coverslips were fixed with 3,7% formaldehyde in phosphate buffered saline (PBS), permeabilized with 0.2% Triton X-100 and blocked with a blocking buffer containing 0.5 g BSA (0163.4, Roth, Karlsruhe, Germany) and 1 ml of FBS (Pan Biotech, Aidenbach, Germany) mixed with 49 ml of PBS. Primary antibody incubation in PBS was performed overnight at 4°C. Negative controls were incubated with only PBS buffer overnight. The next day, coverslips were incubated with the secondary antibody diluted in blocking buffer and were incubated for 1 h at RT. Cell nuclei were stained with 4′,6-Diamidin-2-phenylindol (DAPI) (Roth, Karlsruhe, Germany). Stainings were evaluated using a Leica fluorescence microscope (Leica, Wetzlar, Germany). List of antibodies used for immunocytochemistry is given in Table 2.

**TABLE 2:** List of antibodies used for immunocytochemistry.

Antibody against	Company	Order-no.	Host	Dilution
IBA1	Millipore, Burlington, MA, United States	MABN92	Mouse	1:500
HIF-1α	Novusbio, Centennial, CO, United States	NB100-479	Rabbit	1:100
IL-1β	Cell Signaling, Danvers, MA, United States	12242	Mouse	1:100
Cleaved IL-1β	Thermo Fisher Scientific, Waltham, MA, United States	PA5-105048	Rabbit	1:500
NLRP1 (NALP1)	Cell Signaling, Danvers, MA, United States	4990	Rabbit	1:500
NLRP3	Thermo Fisher Scientific, Waltham, MA, United States	PA5-79740	Rabbit	1:500
NLRC4	Millipore, Burlington, MA, United States	06-1125	Rabbit	1:500
AIM2	Bioss, Woburn, MA, United States	Bs-5986R	Rabbit	1:200
Mouse 488	Thermo Fisher Scientific, Waltham, MA, United States	A-21202	Donkey	1:500
Rabbit 594	Thermo Fisher Scientific, Waltham, MA, United States	A-21207	Donkey	1:500
Mouse 594	Thermo Fisher Scientific, Waltham, MA, United States	A-21203	Donkey	1:500
Rabbit 488	Thermo Fisher Scientific, Waltham, MA, United States	A-21206	Donkey	1:500

### Zone Exclusion Assay

Migration of HMC-3 cells was observed over 48 h 70 μl of 500,000 cells/ml were seeded 1 day prior to the experiment into 2 well culture-inserts (80209, Ibidi GmbH, Gräfelfing, Germany). On the day of experiment the medium was changed, and cells were subjected to either 4 h of OGD (0% FBS, 0.5% P/S) or normoxia (5% FBS, 0.5% P/S) and treated with either 1 μl/ml NaCl or 1 U/ml rhEPO. Afterward inlets were removed, creating a 500 μm sized gap between both cell monolayers. Medium was changed to 5% RPMI with 0.5% P/S and an additional 10 μg/ml Mitomycin C (10107409001, Roche, Germany). Mitomycin C served as a proliferation inhibitor. Cells were monitored using live cell imaging (Carl Zeiss, Oberkochen, Germany).

### ROS Assay

The ROS assay was performed according to the manufacturer’s protocol. In brief, after OGD-stimulation 5 μM CellROX™ (C10444, Thermo Fisher Scientific, Waltham, MA, United States) reagent was added to the medium. After 30 min of incubation at 37°C the supernatant was removed, and the cells were washed three times with PBS. Fluorescence was measured at an excitation wavelength of 488 nm and emission wavelength of 525 nm.

### Reverse Transcription Quantitative PCR

Gene expression analysis was performed with human microglial HMC-3 cells. After dissolving and homogenizing cells in PegGold (PeqLab #30-2010, Erlangen, Germany) total RNA was extracted using peqGold RNA TriFast as previously described (Zeyen et al., 2020). Complementary DNA was synthesized using an iScript™ cDNA Synthesis Kit (Bio-Rad Laboratories, CA, United States) and random hexanucleotide primers (Invitrogen, Karlsruhe, Germany) using 1 μg of total RNA according to the manufacturer’s protocol. Triplicates of every sample were transferred by a pipetting robot (Corbett CAS-1200, Qiagen, Hilden, Germany) to Rotor-Gene strip reaction tubes (I1402-0400, StarLab, Hamburg, Germany) and RT-qPCR analysis was performed using the Rotor-Gene Q device (Qiagen, Hilden, Germany). RNase free H_2_O (Merck, 64293, Darmstadt, Germany) served as no template control (NTC) and primer efficiencies were calculated using the Pfaffl method (Pfaffl, 2001). The target genes and housekeeping gene (glyceraldehyde-3-phosphate dehydrogenase, *GAPDH*) were measured at cycle threshold (*C*t values), and relative quantification was calculated by the ΔΔCt method using the qbase+ software (Biogazelle, Belgium). The following forward (fwd) and reverse (rev) primers were used (5′- > 3′): *GAPDH* (fwd: CCT GCA CCA CCA ACT GCT TA, rev: GGC CAT CCA CAG TCT TCT CAG), *AIM2* (fwd: TGT GGC TGC TAG TGA GAA CC, rev: GAC ACT TCT GGA CGG CTT CA), *NLRP3* (fwd: CTT CTC TGA TGA GGC CCA AG, rev: GCA GCA AAC TGG AAA GGA AG) *NLRC4* (fwd: GTC TGA CTG ACA GCT TGG GT, rev: AGG TTT TTC AGG CCT TCA GCT A) *NLRP1* (fwd: ATT GAG GGC AGG CAG CAC AGA T, rev: CTC CTT CAG GTT TCT GGT GAC C) *CASP1* (fwd: TTG AGC AGC CAG ATG GTA GAG, rev: TCT TCA CTT CCT GCC CAC AG) *IL-1β* (fwd: CTT CGA GGC ACA AGG CAC AA, rev: TTC ACT GGC GAG CTC AGG TA) *GSDMD* (fwd: ATG AGG TGC CTC CAC AAC TTC C, rev: CCA GTT CCT TGG AGA TGG TCT C).

### Statistics

We utilized GraphPad Prism (version 8.4.3, San Diego, CA, United States) for data analysis and visualization. Residuals were analyzed for normal distribution using the Shapiro–Wilk and D’Agostino–Pearson omnibus normality test. Variance homogeneity was tested using the Bartlett test or the Spearman’s rank correlation test for heteroscedasticity. We utilized a ROUT test to identify outliers. In case of significances in normality and/or variance homogeneity, values were BOX-COX-transformed after calculation of the optimal lambda and used for statistical analysis. Intergroup differences were tested by ANOVA two-way or three-way followed by Tukey *post hoc* test (multiple groups). Data are given as arithmetic means ± SD. *P* < 0.05 was considered statistically significant. The number of experimental and technical repeats are indicated in the corresponding figure legends including the statistical tests employed.

## Results

### Administration of Recombinant Human Erythropoietin Mitigated Oxygen-Glucose-Deprivation-Induced Cell Death and Impairment of Metabolism in Human Microglial HMC-3 Cells

**FIGURE 1:**
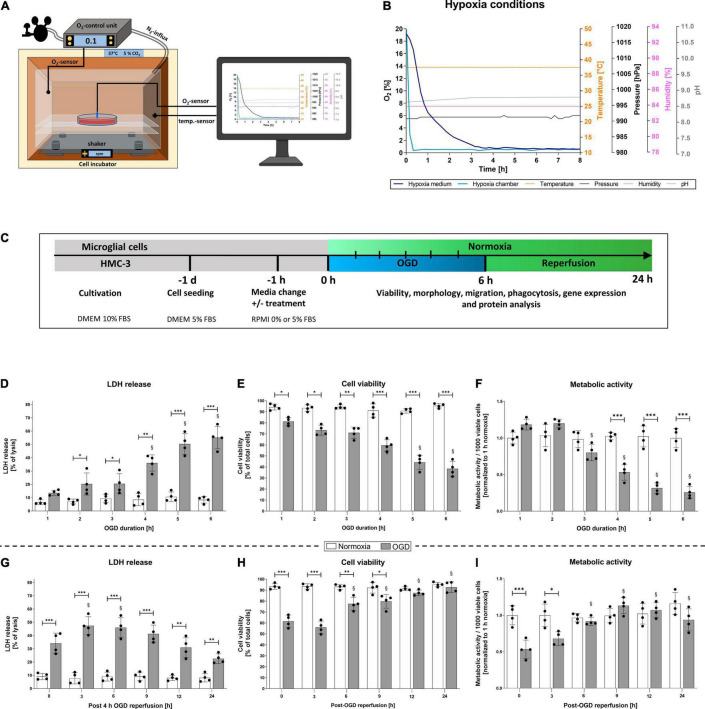
Increasing oxygen-glucose-deprivation (OGD) periods enhance cell death rates and decrease the metabolic activity of human HMC-3 microglial cells. **(A)** Schematic illustration of customized hypoxia chamber with the ability to monitor and control environmental parameters such as oxygen levels, temperature, pressure, humidity and pH value in the media. **(B)** Measurement of environmental parameters during HMC-3 OGD experiments (*n* = 4 independent experiments). **(C)** Workflow of OGD and reperfusion (OGD/R) protocol in HMC-3 cells. **(D)** LDH release, **(E)** cell count, and **(F)** metabolic activity after 1–6 h of OGD or the corresponding normoxia control. An OGD duration of 4 h was established for subsequent experiments. **(G)** LDH release, **(H)** cell count, and **(I)** metabolic activity after 4 h of OGD or normoxia followed by up to 24 h of reperfusion. Intergroup differences were tested by ANOVA two-way followed by Tukey *post hoc* test (multiple comparison). Bars represent means ± SD of 4 individual experiments with 3 technical replicates in each experiment, individual data points are shown. *p < 0.05, ^**^p < 0.01, ^***^p < 0.001 between groups, ^§^*p* < 0.05 compared to shortest OGD or reperfusion duration.

**FIGURE 2:**
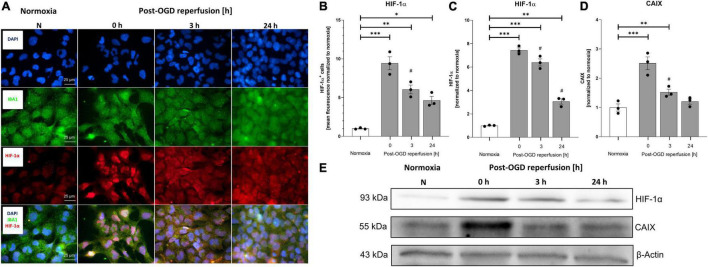
Oxygen-glucose-deprivation (OGD) dependent stabilization of HIF-1α and upregulation of its downstream CAIX in human HMC-3 microglial cells. HMC-3 cells were subjected to 4 h of OGD followed by a reperfusion period of up to 24 h. **(A)** Representative immunocytochemistry images of DAPI (blue), IBA1 (green), and HIF-1α (red) in HMC-3 cells from 3 independent experiments. **(B)** Mean fluorescence intensity per 1,000 cells of HIF-1α. Intergroup differences were tested by ANOVA two-way followed by Tukey *post hoc* test (multiple comparison). Bars represent means ± SD of 3 individual experiments with 3 technical replicates, individual data points are shown. *p < 0.05, ^**^p < 0.01, ^***^p < 0.001 between groups, ^#^*p* < 0.05 compared to previous reperfusion duration. **(C,D)** Densitometric quantification of protein levels normalized to normoxia control. β-Actin served as loading control. Intergroup differences were tested by ANOVA two-way followed by Tukey *post hoc* test (multiple comparison). Bars represent means ± SD of 3 individual experiments, individual data points are shown. *p < 0.05, ^**^p < 0.01, ^***^p < 0.001 between groups, ^#^*p* < 0.05 compared to previous reperfusion duration. **(E)** Representative Western blot images of HIF-1α, CAIX, and β-Actin protein levels.

To mimic ischemia *in vitro* we induced oxygen-glucose-deprivation (OGD) in a self-constructed hypoxia chamber and allowed various post-OGD reperfusion time-points (R) (Figure 1A). Our OGD/R paradigm guaranteed stable and reproducible conditions during experiments by controlling the temperature, pressure, humidity, and pH in the media and by monitoring O_2_-levels in the chamber as well as in the cell media (Figure 1B). The protocol of OGD/R induction in HMC-3 microglial cells is depicted in Figure 1C. We first confirmed that HMC-3 are microglial cells utilizing an IBA staining (Figure 2A). Next, we subjected HMC-3 cells to 1–6 h of OGD to assess the direct effect of OGD on cell viability and metabolic activity in order to establish an OGD duration suitable for further experiments (Figure 1C). An increase of OGD duration enhanced the release of LDH along with elevated rates of cell death, while up to 3 h of OGD had no impact on the metabolic activity of HMC-3 cells (Figures 1D,E). However, a duration beyond 3 h of OGD significantly reduced metabolic activity compared to normoxia controls (*p* < 0.001) (Figure 1F). In addition, upon 4 h of OGD we reached severe hypoxic conditions (O_2_ < 1%) in the medium and could observe significant differences compared to normoxia controls in all three assays (Figure 1B). We therefore decided to use 4 h of OGD as a stimulus for further experiments, as this ensured a sufficient hypoxic stimulus. Reperfusion following 4 h of OGD showed increased levels of LDH after a reperfusion period of 3 h, which steadily declined during later time points (*p* < 0.05) (Figure 1G). However, no significant decrease in cell viability during reperfusion was evident (Figure 1H). Cell viability post-OGD continuously increased over time and was comparable to normoxia control after 12 h of reperfusion while metabolic activity reached the levels of the corresponding normoxia control after 6 h of reperfusion (Figures 1H,I). We further validated the sufficiency of our hypoxic stimulus by examining the stabilization of the intracellular oxygen sensor HIF-1α and its downstream target CAIX (Figure 2). Immunocytochemical (ICC) staining and Western blot analysis exhibited a significantly higher stabilization of HIF-1α after 4 h of OGD compared to normoxia (Figures 2A–C). Quantification of ICC staining and Western blot revealed significantly elevated HIF-1α protein levels following OGD compared to normoxia control (*p* < 0.001), which remained elevated up to 24 h following OGD (*p* < 0.05) (Figures 2B,C). Similarly, protein levels of CAIX peaked directly after 4 h of OGD but remained elevated only until 3 h of reperfusion (Figure 2D).

**FIGURE 3:**
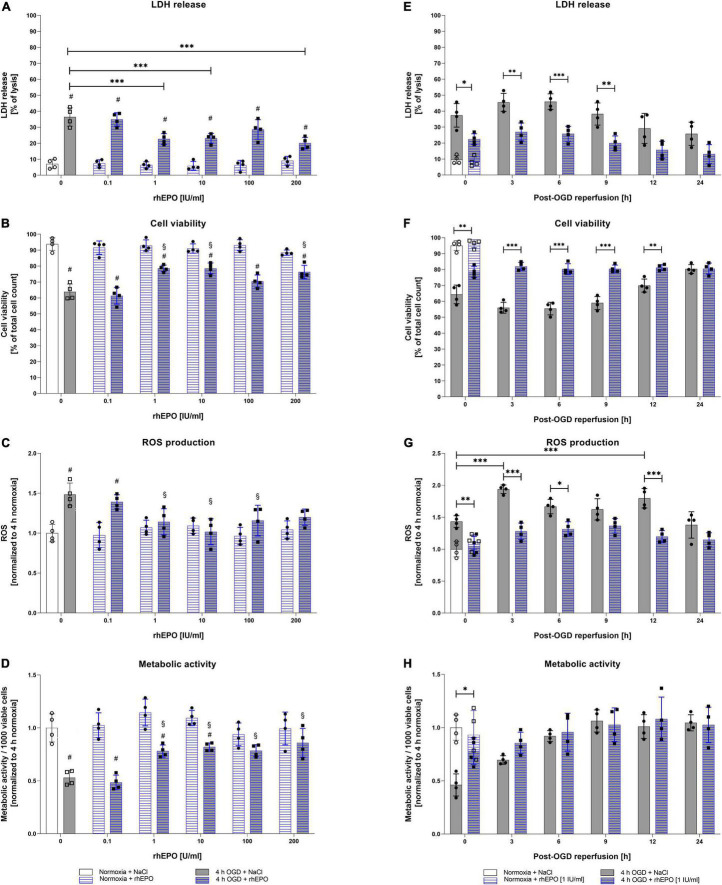
Human recombinant erythropoietin (rhEPO) mitigates OGD-induced cell death, ROS production, and enhances metabolic activity in HMC-3 microglial cells. Dose-dependent impact of rhEPO [0.1–200 IU/ml] on **(A)** LDH release, **(B)** cell count, **(C)** ROS production, and **(D)** metabolic activity of HMC-3 microglial cells after 4 h of OGD or the corresponding normoxia control. Impact of a single-dose rhEPO [1 IU/ml] on **(E)** LDH release, **(F)** cell count, **(G)** ROS production and **(H)** metabolic activity after 4 h of OGD or normoxia followed by 24 h of reperfusion. Saline served as vehicle control (0.9% NaCl). Intergroup differences were tested by ANOVA two-way or three-way followed by Tukey post-hoc test (multiple groups). Bars represent means ± SD of 4 individual experiments with 3 technical replicates in each experiment, individual data points are shown. *p < 0.05, ^**^p < 0.01, ^***^p < 0.001 between groups, ^§^*p* < 0.05 compared to saline control. ^#^*p* < 0.05 compared to normoxia group.

Following the assessment of OGD/R-tolerance of HMC-3 cells, we next aimed to evaluate the dose-dependent impact of rhEPO on these cells. Thus, we subjected HMC-3 cells to 4 h of OGD and administered a single-dose of rhEPO [0.1–200 IU/ml] to subsequently measure its impact on LDH release, cell viability, metabolic activity, and ROS production. Single-dose of 0.1–200 IU/ml rhEPO showed no impact on LDH release, cell viability, cell metabolism and ROS production under normoxic conditions (Figures 3A–D). However, rhEPO significantly decreased post-OGD LDH release compared to vehicle control (*p* < 0.001) when applied at a concentration of 1, 10, and 200 IU/ml, while 100 IU/ml displayed only a tendency of LDH reduction (*p* = 0.1543) (Figure 3A). Similarly, 1, 10, and 200 IU/ml of rhEPO increased cell viability compared to vehicle control (*p* < 0.01, Figure 3B). ROS production was significantly increased after 4 h of OGD (*p* < 0.01, Figure 3C). In the course of reperfusion, ROS production was further increased peaking after 3 h of reperfusion (*p* < 0.001) (Figure 3G). Single-dose of 1, 10, and 100 IU/ml significantly decreased ROS production after 4 h of OGD (*p* < 0.05), while a rhEPO concentration of 200 IU/ml did not reduce ROS levels compared to saline control (Figure 3C).

Metabolic activity after 4 h of OGD was increased after a single dose administration of rhEPO in the range of 1 – 200 IU/ml compared to saline control (*p* < 0.05, Figure 3D). Overall, 1 IU/ml of rhEPO appeared to induce a notable reduction in LDH release, ROS production, and the accompanied cell death after OGD, while significantly increasing metabolic activity. We therefore chose a single-dose of 1 IU/ml rhEPO for further experiments. Effects of single-dose administration of 1 IU/ml rhEPO after 4 h of OGD followed by a reperfusion period of up to 24 h revealed a significant decrease in LDH release up to 9 h following OGD (Figure 3E). In line with a decrease in LDH release, cell viability was also increased up to 12 h following reperfusion (Figure 3F). Administration of rhEPO positively affected the metabolic activity of HMC-3 cells and significantly decreased ROS levels compared to the vehicle control in the course of post-OGD reperfusion (Figure 3H).

These data suggest that rhEPO possesses cytoprotective properties, as it was able to significantly reduce OGD/R induced LDH release, cell death, ROS production and metabolic activity.

### Single-Dose of Recombinant Human Erythropoietin Increased Cell Migration and Restored Phagocytic Activity After Oxygen-Glucose-Deprivation

**FIGURE 4:**
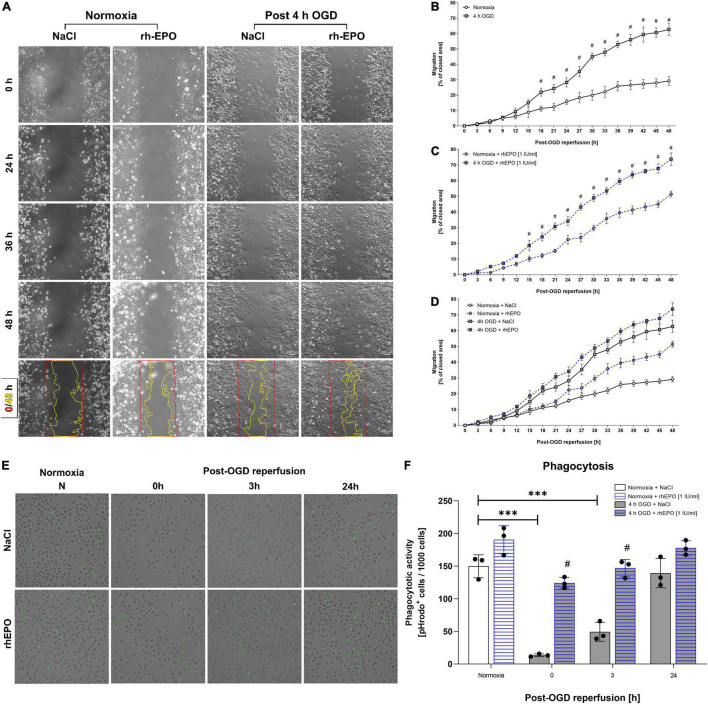
Administration of recombinant human erythropoietin (rhEPO) increases migration and restores OGD-dependent decrease of phagocytic activity in HMC-3 microglial cells. HMC-3 microglial cells were subjected to 4 h of OGD treated with a single-dose of rhEPO [1 IU/ml] or NaCl. Migration ability was assessed using a zone exclusion assay. **(A)** Representative images of HMC-3 migration over a period of 48 h after stimulation. Quantification of HMC-3 migration treated either with **(B)** NaCl or **(C)** rhEPO [1 U/ml] after 4 h of OGD or corresponding normoxia control. **(D)** Overview of migration of both NaCl and rhEPO [1 U/ml] treated cells. Intergroup differences were tested by multiple *t*-tests followed by two-stage set-up method of Benjamini, Krieger, and Yekutieli. Data are presented as means ± SD of 3 individual experiments with 3 technical replicates in each experiment. ^#^*p* < 0.05 compared to normoxia control. **(E)** Representative images of phagocytic activity of HMC-3 after 4 h OGD using pHrodo™ Green *E. coli* Bioparticles.™ **(F)** Quantification of phagocytic activity. pHrodo™ Green *E. coli* Bioparticles™ positive cells per 1,000 counted cells were quantified in each experiment. Intergroup differences were tested by ANOVA two-way followed by Tukey *post hoc* test (multiple comparison). Bars represent means ± SD of 3 individual experiments with three technical replicates in each experiment, individual data points are shown. *p < 0.05, ^**^p < 0.01, ^***^p < 0.001 between groups, ^#^*p* < 0.05 compared to vehicle group.

To assess the impact of OGD/R and rhEPO on HMC-3 cell functionality, we evaluated migration and phagocytosis of HMC-3 microglial cells upon 4 h of ODG followed by a reperfusion period of 48 h (Figures 4A,E). ODG appeared to increase cell migration compared to vehicle control in the course of reperfusion (*p* < 0.05, Figure 4B). A single dose of rhEPO markedly increased cell migration after both normoxia and ODG (*p* < 0.05, Figure 4C). Both OGD and rhEPO enhanced the migration capacity of HMC-3 microglial cells (Figure 4D). Notably, 4 h of OGD as well as a single-dose of rhEPO appeared to influence cell migration beyond the acute phase, since we observed a steady increase in migration throughout the whole observation period of 48 h.

In addition to migration, we sought to assess the impact of OGD/R and rhEPO administration on microglial phagocytosis. HMC-3 cells displayed almost no phagocytic activity following OGD (Figures 4E,F). This impairment of phagocytic activity remained for 3 h post-OGD reperfusion and was restored to basal levels after 24 h of reperfusion. Upon administration of rhEPO, HMC-3 cells exhibited no decrease in phagocytic activity after OGD and subsequent reperfusion.

In conclusion, our data therefore suggest, that rhEPO enhances microglial migration and counteracts OGD/R-induced impairment of phagocytosis.

### Oxygen-Glucose-Deprivation-Mediated Upregulation and Activation of the NLRP1, NLRP3, AIM2, and NLRC4 Inflammasomes as Well as the Associated Downstream Cascade Were Dampened by Recombinant Human Erythropoietin

**FIGURE 5:**
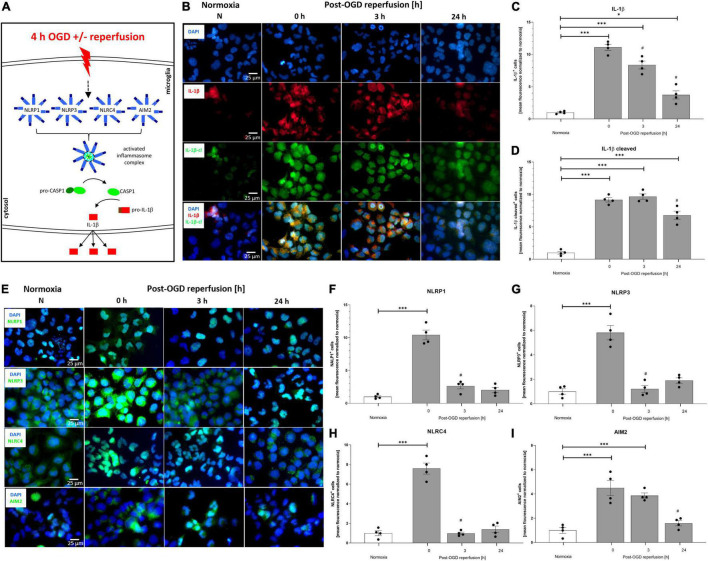
Oxygen-glucose-deprivation-dependent upregulation of IL-1β and the NLRP1, NLRP3, NLRC4, and AIM2 inflammasomes in human microglial cells. HMC-3 microglial cells were subjected to 4 h of OGD followed by a reperfusion period of up to 24 h. **(A)** Schematic illustration of the inflammasome cascade upon OGD. Representative immunocytochemistry of **(B)** DAPI (blue), IL-1β (red), cleaved IL-1β (green), and **(E)** NLRP1, NLRP3, NLRC4, and AIM2 (all inflammasomes green) from 3 independent experiments. Mean fluorescence intensity per 1,000 cells of **(C)** IL-1β, **(D)** cleaved IL-1β and **(F)** NLRP1, **(G)** NLRP3, **(H)** NLRC4, and **(I)** AIM2. Intergroup differences were tested by ANOVA two-way followed by Tukey *post hoc* test (multiple comparison). Bars represent means ± SD of 3 individual experiments with 3 technical replicates, individual data points are shown. *p < 0.05, ^**^p < 0.01, ^***^p < 0.001 between groups, ^#^*p* < 0.05 compared to previous reperfusion duration.

To delineate the impact of OGD/R and rhEPO administration on the regulation and activation of the NLRP1, NLRP3, NRLC4, and AIM2 inflammasomes, we first analyzed their indirect cleavage product IL-1β (schematic illustration of inflammasome mediated IL-1β cleavage by CASPASE1 is depicted in Figure 5A). ICC staining revealed increased IL-1β and cleaved IL-1β protein levels after 4 h of OGD (Figure 5B). Quantification of mean fluorescence intensity showed elevated IL-1β protein-levels directly after OGD, which remained increased up to 24 h post OGD (Figure 5C). Cleaved IL-1β protein revealed the highest levels 3 h post-OGD (Figure 5D). The NLRP1, NLRP3, NLRC4, and AIM2 inflammasomes appeared to be present in HMC-3 microglial cells as demonstrated by ICC-staining (Figure 5E). The mean fluorescence intensity of all four inflammasomes was significantly increased directly after OGD and dropped to the normoxic control levels after 3 h of reperfusion (Figures 5F–I).

**FIGURE 6:**
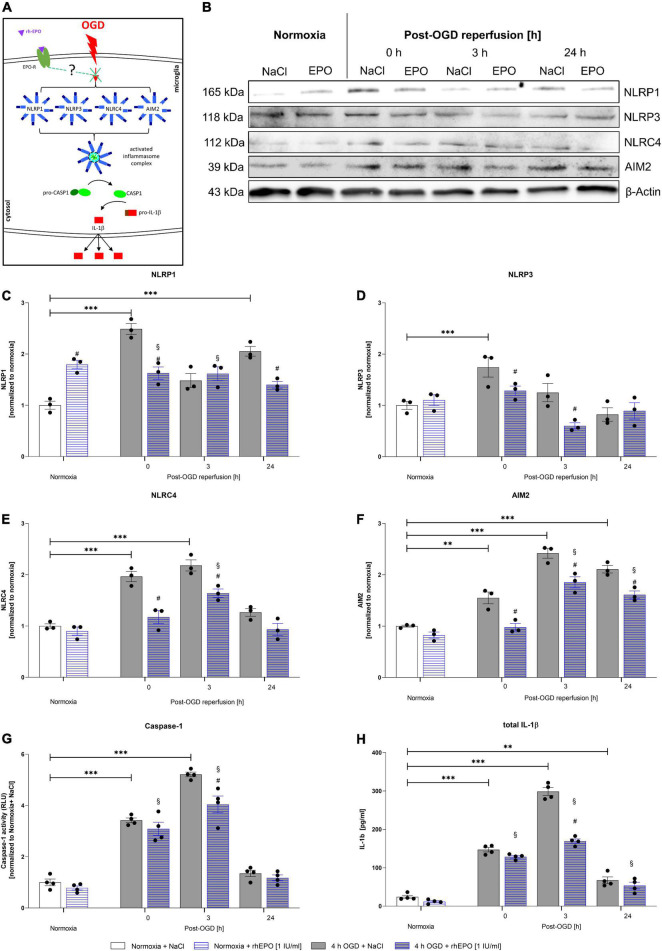
Recombinant human erythropoietin abrogates an upregulation of the NLRP1, NLRP3, NLRC4, and AIM2 inflammasomes and of CASPASE1 and IL-1β protein levels after 4 h of OGD followed by a reperfusion period of 24 h. HMC-3 cells were treated with a single-dose of rhEPO [1 IU/ml] prior to 4 h of OGD followed by a reperfusion period of (up to) 24 h. Saline served as vehicle control (0.9% NaCl). **(A)** Schematic illustration addressing the potential impact of rhEPO and OGD on the activation of inflammasomes. **(B)** Representative Western blot images of NLRP1, NLRP3, NLRC4, AIM2, and β-Actin protein levels. **(C–F)** Densitometric quantification of protein levels normalized to saline normoxia control. β-Actin served as loading control. Intergroup differences were tested by ANOVA two-way followed by Tukey *post hoc* test (multiple comparison). Bars represent means ± SD of 3 individual experiments, individual data points are shown. *p < 0.05, ^**^p < 0.01, ^***^p < 0.001 between groups, ^§^*p* < 0.05 compared to normoxia + NaCl. ^#^*p* < 0.05 compared to vehicle group. **(G)** CASPASE1 activity was assessed by CASPASE1-Glo^®^ assay and normalized to normoxia saline control. **(H)** IL-1β concentrations [pg/ml] in the supernatant of HMC-3 cells were determined by ELISA. Bars represent means ± SD of 4 individual experiments with 3 technical replicates in each experiment, individual data points are shown. *p < 0.05, ^**^p < 0.01, ^***^p < 0.001 between groups, ^§^*p* < 0.05 compared to normoxia + NaCl. ^#^*p* < 0.05 compared to vehicle group.

We next aimed to examine the effect of OGD/R and rhEPO on the regulation of the NLRP1, NLRP3, NLRC4, and AIM2 inflammasome sensors and the activation of their downstream cascade (Figure 6A). OGD and up to 3 h of reperfusion significantly elevated mRNA levels of all four inflammasomes as well as *CASPASE1*, *IL-1β*, and *GSDMD* compared to normoxia controls (*p* < 0.05, Supplementary Figures 1A–G). Protein levels of all four inflammasome sensors were significantly elevated after OGD compared to normoxia controls (*p* < 0.05, Figures 6C–F). NLRP3 protein levels quickly returned to normoxic levels during reperfusion, whereas NLRC4 remained elevated up to 3 h of reperfusion and NLRP1 and AIM2 even after 24 h of reperfusion (Figures 6C–F). Similarly, the activation of CASPASE1, maturation of total IL-1β, GSDMD and GSDMD-N protein levels displayed a significant increase following OGD and reaching their maximum at 3 h after OGD (*p* < 0.001, Figures 6G,H) (*p* < 0.01, Supplementary Figures 1H,J,K) (representative images of WB shown in Supplementary Figure 1I). During the same observation period, however, the increase in pro-IL1β compared to normoxia was distinctly lower than that of total IL-1-β (*p* < 0.01, Supplementary Figures 2A,B). Application of rhEPO dampened OGD-induced mRNA and protein expression levels of inflammasomes and respective downstream events (Supplementary Figures 1A–G) (representative images of WB shown in Figure 6B).

The effect of rhEPO could also be observed after 3 h of reperfusion in the NLRP3, NLRC4, and AIM2 inflammasome sensors. At the latest time-point of observation (24 h post-OGD), NLRP1 and AIM2 were found to be decreased compared to vehicle control. The effect of rhEPO on CASPASE1 was apparent directly after OGD and after 3 h of reperfusion, while rhEPO significantly reduced total IL-1β levels after 3 h of reperfusion. At the same time rhEPO had no significant impact on the protein levels of pro-IL-1β and GSDMD in our ELISA (Supplementary Figures 1H, 2B,D). It should be noted, however, that the application of rhEPO exerted a mitigating effect in GSDMD protein levels (*p* < 0.0217, Supplementary Figure 1H) and showed a mitigating tendency in GSDMD-N protein levels after 3 h of reperfusion on Western blot (*p* < 0.0521, Supplementary Figures 1I–K).

In conclusion, OGD/R induced the activation of the NLRP1, NLRP3, NLRC4, and AIM2 inflammasomes in human microglial HMC-3 cells. Administration of rhEPO diminished the activation of the four canonical inflammasomes suggesting that EPO-conveyed cytoprotective and anti-inflammatory properties might be mediated via the regulation of inflammasomes.

### Functional Inhibition of the NLRP3 Inflammasome via MCC950 Did Not Alter IL-1β Maturation Rates After Oxygen-Glucose-Deprivation/Reperfusion

**FIGURE 7:**
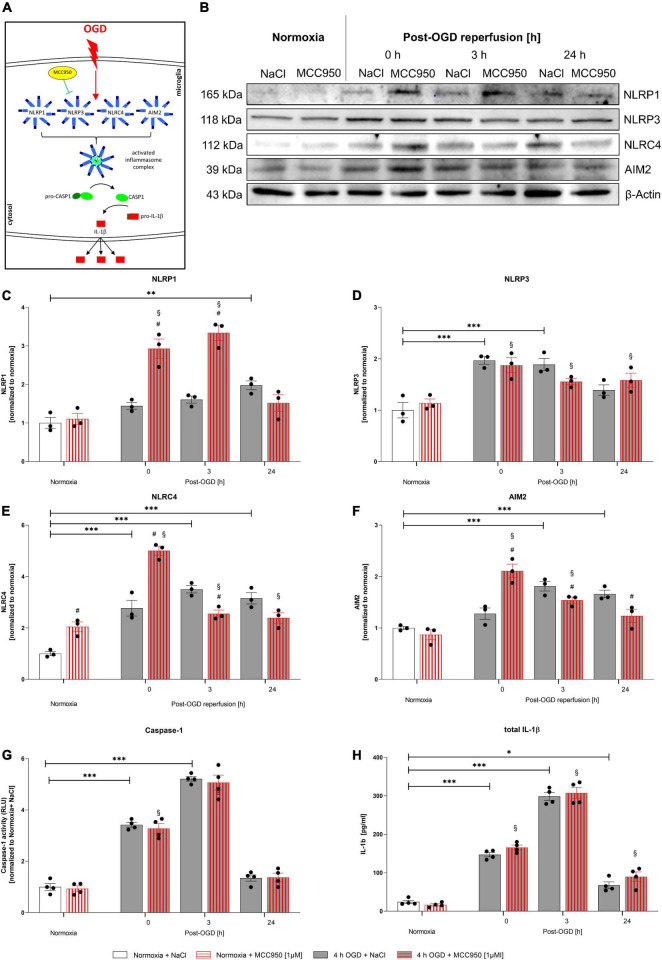
Functional inhibition of NLRP3 inflammasome using MCC950 induces an upregulation of the NLRP1, NLRC4, and AIM2 inflammasomes after 4 h of OGD followed by a reperfusion period of 24 h. HMC-3 cells were treated with a single-dose of MCC950 [1 μM] and subjected to 4 h of OGD followed by a reperfusion period of up to 24 h. **(A)** Schematic illustration of functional inhibition of the NLRP3 inflammasome upon OGD. **(B)** Representative Western blot images of NLRP1, NLRP3, NLRC4, AIM2, and β-Actin protein levels. **(C–F)** Densitometric quantification of protein levels normalized to saline normoxia control. β-Actin served as loading control. Intergroup differences were tested by ANOVA two-way followed by Tukey *post hoc* test (multiple comparison). Bars represent means ± SD of 3 individual experiments, individual data points are shown. *p < 0.05, ^**^p < 0.01, ^***^p < 0.001 between groups, ^§^*p* < 0.05 compared to normoxia + NaCl. ^#^*p* < 0.05 compared to vehicle group. **(G)** CASPASE1 activity was assessed by CASPASE1-Glo^®^ assay and normalized to normoxia saline control. **(H)** IL-1β concentrations [pg/ml] in the supernatant of HMC-3 cells were determined by ELISA. Bars represent means ± SD of 4 individual experiments with 3 technical replicates in each experiment, individual data points are shown. *p < 0.05, ^**^p < 0.01, ^***^p < 0.001 between groups, ^§^*p* < 0.05 compared to normoxia + NaCl. ^#^*p* < 0.05 compared to vehicle group.

In order to assess, whether the NLRP3 inflammasome is the main inflammasome in microglial cells executing the inflammatory response after OGD/R, we utilized MCC950 to functionally inhibit the NLRP3 inflammasome (Figure 7A). MCC950 administration had no impact on CASPASE1 activity and total IL-1β release (Figures 7G,H). However, MCC950 appeared to affect the NLRP1, NLRC4, and AIM2 protein levels after OGD/R (representative WB images are shown in Figure 7B). NLRP1, NLRC4, and AIM2 protein levels were significantly increased after OGD when MCC950 was administered, whereas NLRP3 protein levels remained at the same level as the vehicle control (Figures 7C–F). Furthermore, functional inhibition of NLRP3 using MCC950 had no effect on cell viability neither under normoxic conditions nor after 4 h of OGD. Additionally, MCC950 did not alter the cytoprotective effect of rhEPO on cell viability (Supplementary Figure 3).

In conclusion, this data suggests that functional inhibition of NLRP3 is compensated by an upregulation of NLRP1, NLRC4, and AIM2 giving more insight into the processes of IL-1β maturation after OGD/R.

## Discussion

In this study, we for the first time characterized the ischemic tolerance and post-ischemic functional activation of the rarely described human microglial cell line HMC-3. Furthermore, we delineated the impact of rhEPO on microglial functionality and the regulation of the inflammasomes after *in vitro* ischemia. We demonstrated that administration of rhEPO mitigated OGD/R-induced oxidative stress and cell death. In addition, rhEPO enhanced metabolic activity, migration, and phagocytosis of HMC-3 microglial cells after OGD/R. The post-ischemic regulation of the NLRP1, NLRP3, NLRC4, and AIM2 inflammasome sensors and subsequent CASPASE1 activation and maturation of IL-1β were attenuated after rhEPO application. We speak of maturation as the increased levels of pro-IL-1β after 0 h (*p* = 0.0068) and 3 h of reperfusion (*p* = 0.0039) are not nearly as strong as of total IL-1β and must be due to release of mature IL-1β (Supplementary Figure 2). The increase in pro-IL-1β and can be explained by dead cells releasing their cytoplasmatic pro-IL-1β into the supernatant. In addition, since the maturation and release of IL-1β is described to be dependent or at least temporarily associated with the activation of CASPASE1, we found similar increase patterns of CASPASE1 activity after hypoxia (Andrei et al., 2004; Schroder and Tschopp, 2010; Monteleone et al., 2015; Yaron et al., 2015).

Moreover, pharmacological inhibition of the NLRP3 inflammasome utilizing the small molecule inhibitor MCC950 had no effect on the activation and maturation levels of CASPASE1 and total IL-1β after OGD/R, respectively, but its administration increased protein levels of the NLRP1, NLRC4, and AIM2 inflammasome sensors suggesting a compensatory capacity among inflammasomes.

Since the FDA approved its use for patients with renal diseases over three decades ago, EPO has also been reported to exert multifaceted neuroprotective and neurorestorative actions in addition to its hematopoietic effects in both preclinical and clinical stroke studies. Following the numerous positive studies in stroke models [summarized in a systematic review and meta-analysis by Jerndal et al. (2010)], EPO has also been tested in stroke patients. In a proof of concept pilot study (phase I/II), EPO was found not only to be innocuous to stroke patients but also to significantly reduce stroke volumes within 8 h of the onset of clinical symptoms (Hasselblatt et al., 2006). In the subsequent multicenter study (phase III) including 522 patients, however, EPO failed to show positive effects in almost all outcome parameters (Ehrenreich et al., 2009). Interestingly, a higher mortality rate of patients was observed exclusively when EPO was combined with recombinant tissue plasminogen activator (rtPA). Nevertheless, a subgroup analysis in this study revealed that EPO administration on its own was protective in stroke patients (Ehrenreich et al., 2011). Despite the failed multicenter trial, there is a broad consensus that EPO remains a promising candidate for neuroprotective therapy, but extensive molecular and cellular preclinical investigations are needed prior to any potential clinical use in stroke patients. Although the anti-inflammatory effect of EPO has been reported in several studies, its impact on brain-resident immune cells such as microglia and in particular on microglial inflammasomes remains obscure (Grasso et al., 2002; Fisher, 2003; Kumral et al., 2004; Sättler et al., 2004; Lu et al., 2005; Kanaan et al., 2006; Habib et al., 2019b; Simon et al., 2019). Microglia highly express EPOR and account for 10–20% of all glial cells (Qin et al., 2019; Subhramanyam et al., 2019). We first investigated the dose- and time-dependent impact of OGD/R and EPO on viability and metabolism of HMC-3 microglial cells. Using our customized hypoxia chamber, we detected under controlled and reproducible conditions that severe hypoxia (O_2_ < 1.0%) prevailed in the medium after about 3.5 h, whereas oxygen was almost completely displaced in the chamber after only a few minutes (Figure 1). An OGD duration of 4 h not only resulted in significant LDH release accompanied by cell death and impaired metabolic activity of HMC-3 cells but also exhibited a pronounced stabilization of the intracellular oxygen-sensor HIF-1α and its downstream target CAIX (Figures 1, 2). However, it is important to note that although the CTB assay used for metabolic activity assessment depends on an intact mitochondrial respiratory chain, this assay provides only an indication of the cell metabolism. A combination of CTB with the assessment of NADPH/FADH2 or ATP levels would provide a more accurate picture of metabolic activity and should be used for future studies (Kokott-Vuong et al., 2021). During post-hypoxic reperfusion, HMC3 microglial cells appeared to recover and seemed less stressed, as reflected not only in their metabolic activity and cell death rates but also in the reduced HIF-1α stabilization levels. However, using the same OGD/R paradigm under controlled environmental parameters, the frequently used murine BV-2 microglia showed robust hypoxic activation of cells already after 3 h, suggesting that HMC-3 seem to be more resistant to OGD/R than BV-2 microglia (Habib et al., 2013). If this is due to species differences and/or variations in regulators molecules/pathways in these cell lines needs further analyses. Our data suggest that the administration of rhEPO is capable of mitigating OGD/R-induced cell death, and ROS production and in turn appears to enhance the metabolic activity of HMC-3 microglial cells in a dose-dependent manner (Figure 3). We knew from our previous study that a low dose of EPO was more protective compared to a high dose (5,000 vs. 90,000 IU/kg) after transient ischemia in mice (Komnig et al., 2018). Hence, we evaluated a range of EPO concentrations [0.1–200 IU/ml] used in *in vitro* studies with respect to their cytoprotective effects in HMC-3 cells. We have demonstrated that the low concentrations of rhEPO (1 and 10 IU/ml) proved to be more beneficial than the high concentrations (e.g., 200 IU) by significantly reducing ROS production and cell death rates. The question arises whether endogenous EPO as a downstream of HIF-1α is upregulated after OGD and might contribute to neuroprotection. Indeed, hypoxia/ischemia leads to an upregulation of EPO mRNA levels (Brines and Cerami, 2005; Habib et al., 2019a; Fernandez Garcia-Agudo et al., 2021). Chong et al. (2003), however, reported that endogenous EPO alone is not sufficient to protect neurons from cerebral ischemia, while the administration of rhEPO significantly prevented neuronal cell death after stroke. However, the duration and paradigm of the stimulus appear to play a crucial role in enabling an organism to produce a sufficient amount of endogenous EPO, which may exert sufficient cytoprotection. A most recent study reported that rhEPO decreases the total number of microglia by inhibiting their proliferation and by inducing apoptosis. In line with our data, here rhEPO also dampened microglial activity and metabolism allowing undisturbed differentiation of immature neuronal subpopulations into mature hippocampal neurons (Fernandez Garcia-Agudo et al., 2021). These observations were assumed to be dependent on microglial EPOR and the colony-stimulating factor 1 receptor (CSF1R) system. Of note, in this study, mice were subjected to permanent moderate inspiratory hypoxia (12% O_2_) for 3 weeks. At that time, a reduced number of microglia was detected which was comparable to that obtained after treating mice with rhEPO (5,000 IU/kg intraperitoneal injections every other day for 3 weeks). Since we only examined a cell line after acute severe hypoxia/OGD (O_2_ < 1%) with a maximum reperfusion time of 48 h, we cannot exclude that a longer rhEPO administration over several weeks would also result in microglial apoptosis. In our *in vitro* ischemia model, OGD/R resulted in a marked reduction in phagocytosis activity accompanied by an increase in migration compared to the normoxia control (Figure 4). The phagocytosis rates increased again during post-OGD reperfusion. We had previously observed a similar pattern in murine BV-2 microglial cells and had in parallel detected an increased secretion of pro-inflammatory cytokines (Habib et al., 2013). Administration rhEPO increased migration and restored OGD-dependent decrease of phagocytic activity in HMC-3 microglial cells. In contrast to our model, EPO has been reported to dampen motility of cortical microglia *in vivo* and of microglia/astroglia in mixed cultures 24 h after laser-induced micro injuries (Mitkovski et al., 2015). Moreover, Villa et al. (2003) have suggested that EPO might reduce the recruitment of leukocytes and microglial cells to post-ischemic damaged neurons. The difference between this data and ours could be attributed to the different stimuli and cell type used and to the lack of interaction with other brain cells in our model. Future studies using HMC-3 microglia co-cultured with human neuronal/astroglial cell lines may provide interesting insights into cellular influences on microglial migration after ischemia. Similarly, the aforementioned study observed a regulatory effect of EPO on metabolic activity and superoxide/ROS in their micro injury models. ROS has been attributed a critical role in post-hypoxic/ischemic brain damage and may be partly responsible for the induction of inflammasome mediated pyroptosis (Bonda et al., 2010; Dumont and Beal, 2011; Subirós et al., 2012; Abais et al., 2015). An augmented ROS production has been associated with increased activation of the NLRP3 inflammasome in cardiomyocytes (Martinon, 2010; Qiu et al., 2019). HMC-3 microglia respond to OGD/R in the same manner as murine BV-2, as well as primary rat cortical microglial cells by producing ROS (Habib et al., 2013, 2014; Butturini et al., 2019; Voelz et al., 2021). Whether rhEPO-conveyed reduction of post-OGD ROS-levels also affects inflammasome activation has not been reported in microglia so far. As specialized sentinel cells of the innate immunity, microglia elicit a rapid response to post-ischemic homeostatic perturbations in the brain by promoting a robust neuroinflammation through the secretion of pro-inflammatory mediators such as TNF-α, IL-6, IL-1β, and IL-18 (Villa et al., 2003; Qin et al., 2019). The interleukins IL-1β and IL-18 have been attributed a decisive role in post-ischemic apoptosis and pyroptosis and are maturated by inflammasome activated CASPASE1 (Liu and Quan, 2018; de Vasconcelos and Lamkanfi, 2020). Moreover, NLRP3 and subsequent CASPASE1 activation is coupled with cleavage of GSDMD, further promoting pyroptosis and reinforcing the inflammatory response (Ma et al., 2021). The inflammasome mediated pyroptosis has been described as a key contributor to post-ischemic brain damage (Barrington et al., 2017; Dong et al., 2018; Zhang et al., 2019). We could observe significantly (*p* < 0,01) increased mRNA and protein levels of GSDMD coupled with an increase of GSDMD-N protein levels after 4 h of OGD which remained elevated up to 3 h of reperfusion (Supplementary Figures 1G–K) indicating that pyroptosis is sustaining the cell death observed in HMC-3 cells. In murine microglia Ma et al. (2021) showed that inhibition of the NLRP3 inflammasome using salvianolic acids mitigated neuronal cell death by switching microglial phenotype from M1 toward M2 and thus inhibiting the NLRP3 inflammasome/pyroptosis axis. Administration of rhEPO similarly was able to reduce NLRP3 mRNA and protein levels as well as mRNA levels of GSDMD following 4 h of OGD (Figure 6D and Supplementary Figure 1H). However, while the attenuation of protein levels of GSDMD itself was significant (*p* = 0.0217 and Supplementary Figures 1H,J), the attenuation of protein levels of the cleavage product GSDMD-N was not (*p* = 0.0521, Supplementary Figure 1K). In this context, however, the attenuation of GSDMD-N is presumably due to an attenuation of the protein levels of GSDMD itself, since the ratio between GSDMD-N and GSDMD is not significantly altered by application of rhEPO (Supplementary Figure 1L). Nevertheless, further studies examining the protein levels of the cleavage product GSDMD-N and GSDMD and the possible impact of rhEPO on those are needed in order to rule out a possible effect of rhEPO on GSDMD cleavage and pyroptosis. Thus far NLRP3 has been regarded as the main inflammasome involved in neuroinflammation (Luo et al., 2019). Literature research using PubMed (7th April 2022) for the terms “NLRP1,” “AIM2,” “NLRP3,” and “NLRC4” in combination with “stroke” revealed the large difference between the number of original research articles addressing NLRP3 (327) compared to NLRP1 (16), AIM2 (26), and NLRC4 (15). There are few reports attributing a key role to NLRC4 and AIM2 in the pathology of stroke, and even suggesting that NLRC4- or AIM2-deficiency seems to have a more significant neuroprotective impact on the clinical outcomes compared to NLRP3-deficiency after murine stroke (Fann et al., 2013a; Denes et al., 2015; Poh et al., 2019). However, in human microglia and especially in HMC-3 cells, the influence of EPO on post-ischemic activation and regulation of inflammasomes has not been reported so far. We found markedly increased CASPASE1 activity and IL-1β release after OGD, which are not expressed under normoxic conditions in HMC-3 cells (Figure 5). Concomitantly protein levels of NLRP3, NLRP1, NLRC4, and AIM2 were found to be increased. EPO administration dampened the protein levels of the NLRP1, NLRP3, NLRC4, and AIM2 inflammasome sensors and markedly reduced CASPASE1- activation and the release of mature IL-1β (Figure 6). These findings are in line in line with our most recent publication, in which EPO abrogated post-ischemic activation of the NLRP3, NLRC4, and AIM2 inflammasome in murine microglia in a TAK1-dependent manner (Heinisch et al., 2021). To elucidate whether NLRP3 is the main inflammasome responsible for the post-ischemic pyroptosis, we utilized the small molecule MCC950 to functionally suppress the activation of NLRP3 (Perera et al., 2018). Here, we have not been able to detect any effect on the post-ischemic activation of CASPASE1 levels and on the maturation of IL-1β (Figure 7). However, we detected increased levels of the NLRP1, NLRC4, and AIM2 after OGD, indicating a compensatory capacity among the inflammasomes after ischemia. This assumption was further supported by the fact that the functional inhibition of the NLRP3 inflammasome had no effect on cell viability neither under normoxic nor hypoxic conditions (Supplementary Figure 3).

How exactly rhEPO regulates inflammasome levels remains elusive. We know that the pleiotropic glycoprotein EPO controls cell viability and inflammation through multiple downstream effectors including MAP kinases p38 and JNK, as well as the transcription factor NF-kB (Villa et al., 2003; Lieutaud et al., 2008). NF-κB is known to regulate the transcription and priming of the inflammasome sensors and IL-1β (Takahashi et al., 2015; Cao et al., 2020). Binding of EPO to its receptor (EPOR) leads amongst others to the activation of the JAK2/STAT3 pathway, which results in decreased NF-kBp65 phosphorylation and concomitant suppression of pro-inflammatory cytokines (TNF-a, IL-6, and IL-1β) (Cao et al., 2020; Li et al., 2020). Therefore, we hypothesize that rhEPO-mediated inhibition or reduction of NF-κB phosphorylation might lead to a suppression of the priming of the inflammasomes. Future studies utilizing successive and selective inhibition of the NF-κB p65 cascade in microglia after rhEPO administration should be performed to further elucidate these potential interactions.

## Conclusion

We provide evidence that rhEPO not only mitigated ischemia-induced oxidative stress and cell death but also enhanced metabolic activity, migration and phagocytosis of human HMC-3 microglial cells. In addition, rhEPO attenuated post-ischemic regulation of the NLRP1, NLRP3, NLRC4, and AIM2 inflammasome sensors as well as their downstream effectors CASPASE1 and IL-1β. Our data suggest that EPO might mediate its cytoprotective effects via the regulation of inflammasomes. After characterization of the ischemic tolerance of HMC-3, this microglial cell line of human origin may offer a promising alternative to rodent cell lines in stroke research.

## Data Availability Statement

The original contributions presented in this study are included in the article/Supplementary Material, further inquiries can be directed to the corresponding author.

## Author Contributions

EA: conceptualization, methodology, formal analysis, investigation, data curation, writing – original draft preparation, review and editing, and visualization. OH: validation, investigation, and review and editing. MB: investigation, review and editing, and visualizaton. LG: validation, investigation, and review and editing, and visualization. AS: validation and review and editing. AR and MH: formal analysis and review and editing. JS: formal analysis, resources, and review and editing. TW: validation, and review and editing. PH: conceptualization, methodology, formal analysis, investigation, resources, review and editing, visualization, supervision, project administration, and funding acquisition. All authors contributed to the article and approved the submitted version.

## Conflict of Interest

The authors declare that the research was conducted in the absence of any commercial or financial relationships that could be construed as a potential conflict of interest.

## Publisher’s Note

All claims expressed in this article are solely those of the authors and do not necessarily represent those of their affiliated organizations, or those of the publisher, the editors and the reviewers. Any product that may be evaluated in this article, or claim that may be made by its manufacturer, is not guaranteed or endorsed by the publisher.
