# Supplementary figures and images for: Erythropoietin Enhances Post-ischemic Migration and Phagocytosis and Alleviates the Activation of Inflammasomes in Human Microglial Cells
Source: Front Cell Neurosci. 2022 Jun 24;16:915348. doi: 10.3389/fncel.2022.915348 (PMC9263298; doi:10.3389/fncel.2022.915348)

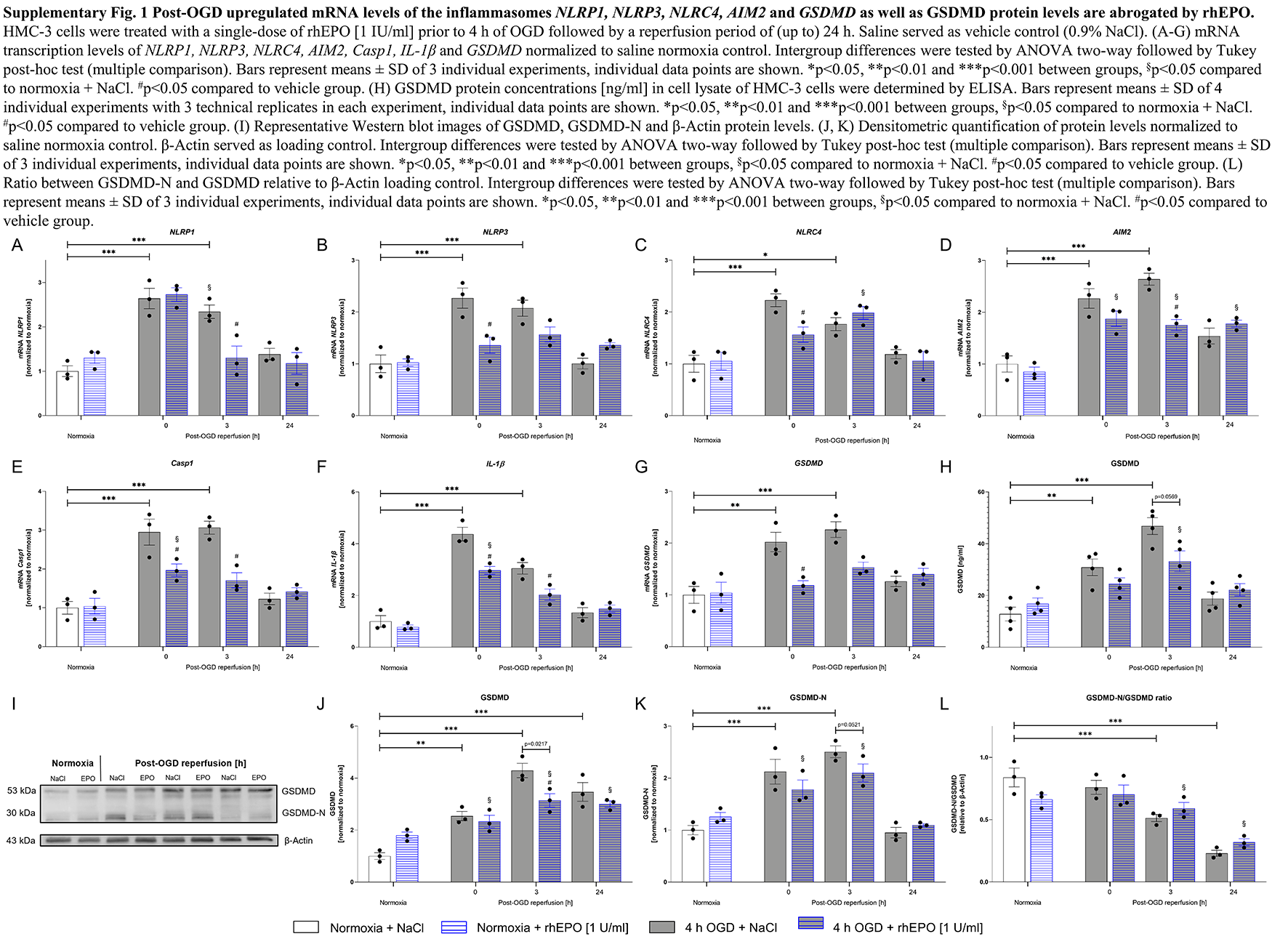

Supplement: Supplementary file 1 [file Image_1.tif]

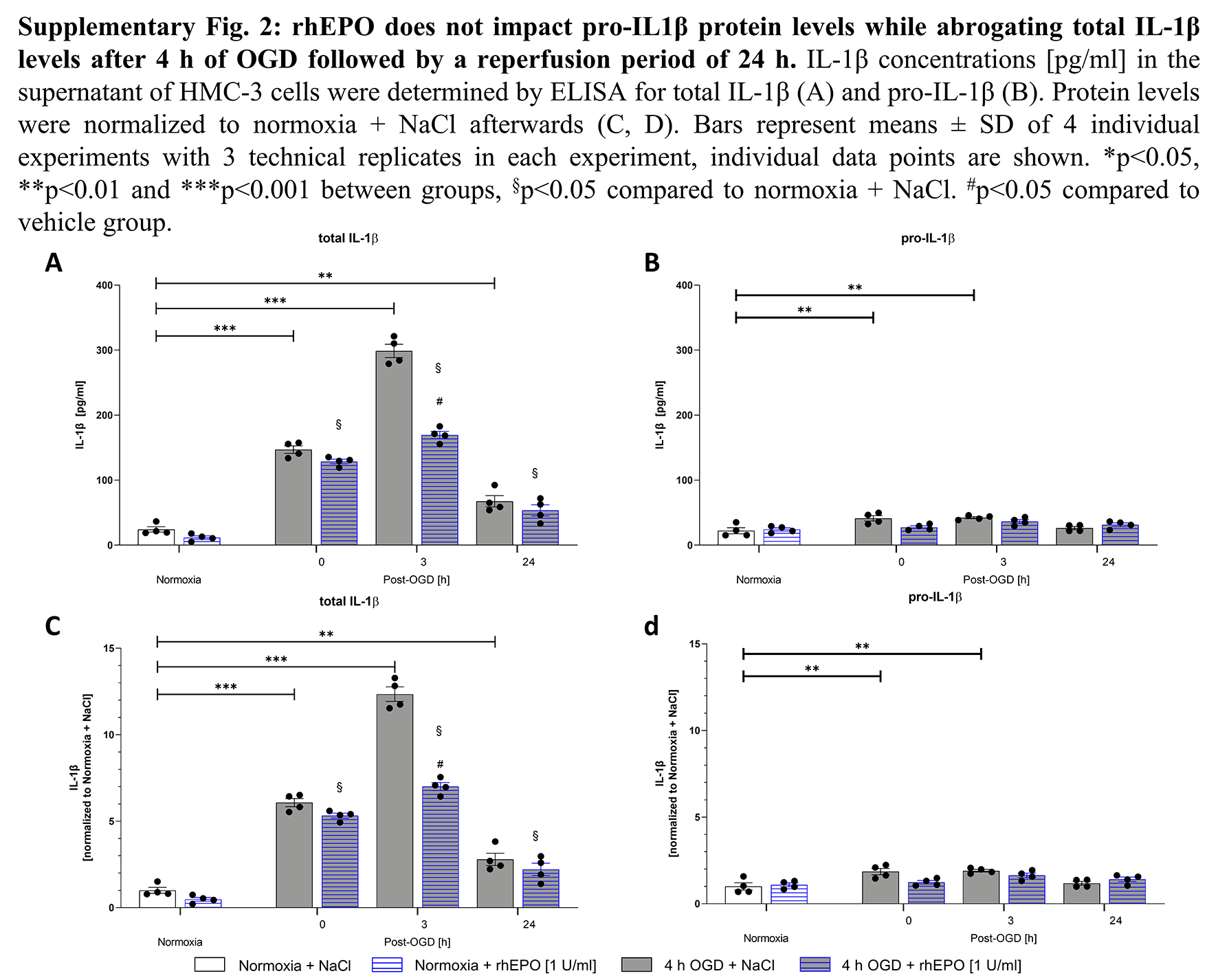

Supplement: Supplementary file 2 [file Image_2.tif]

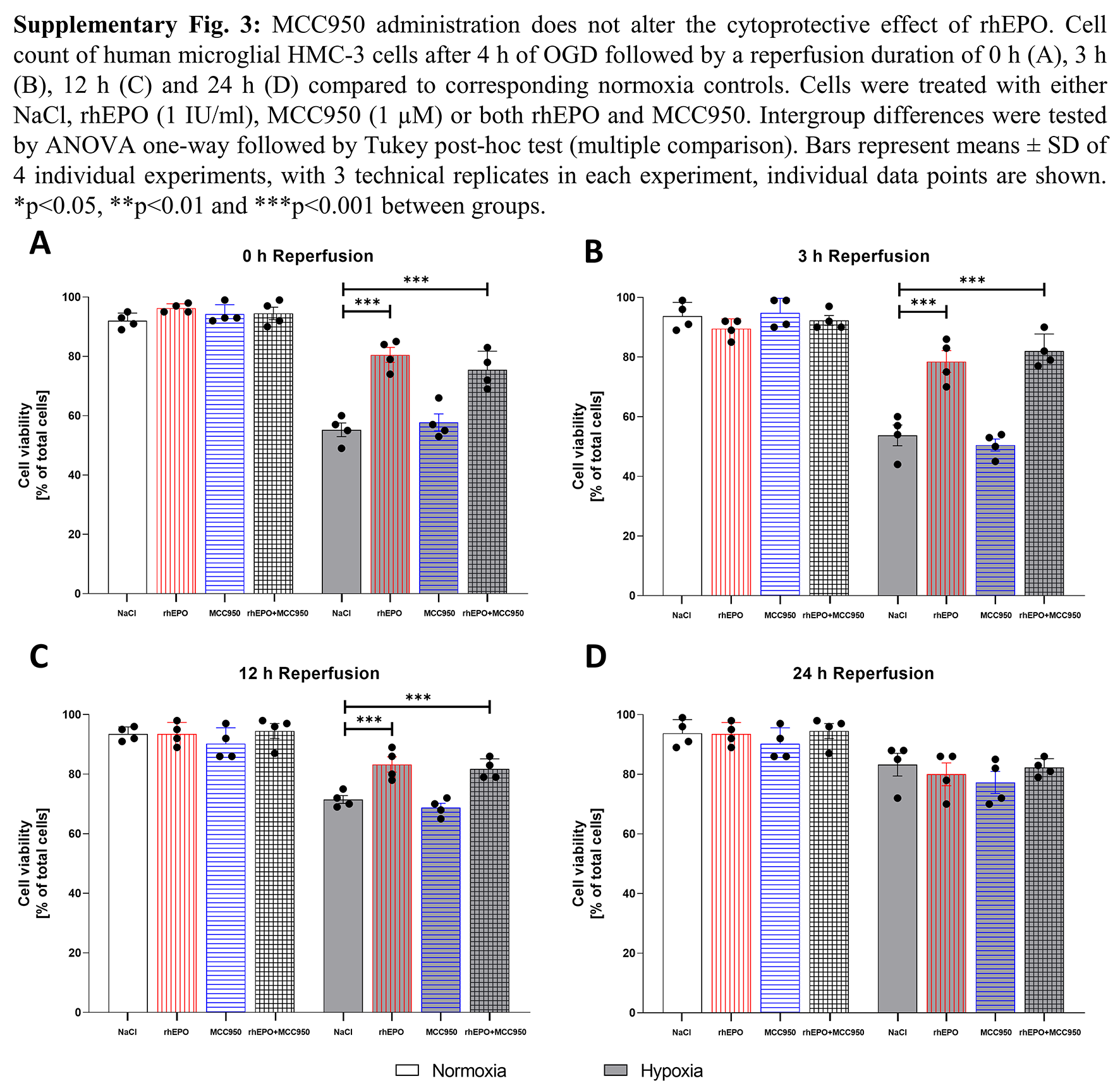

Supplement: Supplementary file 3 [file Image_3.tif]
